# Evaluation by Citation: Trends in Publication Behavior, Evaluation Criteria, and the Strive for High Impact Publications

**DOI:** 10.1007/s11948-015-9638-0

**Published:** 2015-03-06

**Authors:** Maarten van Wesel

**Affiliations:** 1 Department of Education and Research Support, University Library, Maastricht University, PO Box 616, 6200 MD Maastricht, The Netherlands; 2 Department of Family Medicine, Faculty of Health, Medicine and Life Sciences, Maastricht University, PO Box 616, 6200 MD Maastricht, The Netherlands

**Keywords:** Publish or perish, High impact, Research evaluation, Publication practices

## Abstract

Criteria for the evaluation of most scholars’ work have recently received wider attention due to high-profile cases of scientific misconduct which are perceived to be linked to these criteria. However, in the competition for career advancement and funding opportunities almost all scholars are subjected to the same criteria. Therefore these evaluation criteria act as ‘switchmen’, determining the tracks along which scholarly work is pushed by the dynamic interplay of interests of both scholars and their institutions. Currently one of the most important criteria is the impact of publications. In this research, the extent to which *publish or perish*, a long standing evaluation criterion, led to scientific misconduct is examined briefly. After this the strive for high impact publications will be examined, firstly by identifying the period in which this became an important evaluation criterion, secondly by looking at variables contributing to the impact of scholarly papers by means of a non-structured literature study, and lastly by combining these data into a quantitative analysis.

## Introduction

Not ideas, but material and ideal interests, directly govern men’s conduct. Yet very frequently the ‘world images’ that have been created by ‘ideas’ have, like switchmen, determined the tracks along which action has been pushed by the dynamics of interest. ‘From what’ and ‘for what’ one wished to be redeemed and, let us not forget, ‘could be’ redeemed, depended upon one’s image of the world. (Weber 1970b)

Scientific misconduct has been increasing, however until recently awareness of these practices appears to be limited (Regmi 2011).^1^ Recent cases of scientific misconduct, such as the Stapel affaire (fabrication of data, see Levelt Committee et al. 2012), and the Karl-Theodor zu Guttenberg plagiarism affair, have created some awareness amongst both scholars and the general public. This sparked some national or field-specific movements such as *Science in Transition* in the Netherlands (Dijstelbloem et al. 2013) and the *American Society of Cell Biology’s Declaration on Research Assessment* (Moustafa 2014). These movements perceive a link between the system of evaluation of science and cases of scientific misconduct, such as some of the extreme cases mentioned above.

Reflecting on the switchmen metaphor put forth by Weber in his study of religion and its influence on economic systems, we can begin to see that what is deemed as important in science steers the behavior of scholars in a certain direction. Two crucial ideas about what is important in science are currently at work: the idea that publishing more is better and the idea that a journal, paper, scholar and institution should have a high Impact Factor.

*From what* and *for what* does a scholar wish to be redeemed, and *can* a scholar be redeemed? In most western countries a publication track record is necessary for obtaining tenure and it is hard to secure funds for research without one (Regmi 2011). Especially peer-reviewed publications are important for the career of scholars at all stages of employment (Elliott 2013; Moustafa 2014). Someone who only publishes work with a low impact factor will have difficulty obtaining tenure and funds. Thus both the number of publications as well as their impact are crucial for the career of a scholar, *redeeming* him/her from joblessness, or at least careerlessness, if their publication record is *better* than that of their peers. Because when there is stiff competition for positions, funding, and other academic rewards, those with slightly greater achievements will reap in a disproportionately larger share of the rewards (Anderson et al. 2007).

The ideas about what is important in science are important themselves. Scholars are constantly being reminded that these ideas are important, thus these ideas determine the tracks via which action has been pushed by the interest of scholars. This is coupled with a rise of *careerism* among scientists, in which, sometimes, the shortest routes to success are taken, including fraudulent behavior (Kumar 2008) or cutting a few corners (Anderson et al. 2007). Competition between scientists increases the chance of scientific misconduct (Anderson et al. 2007).

First, the literature on *publish or perish* will be examined as it has already been established how this idea has shaped scholarly publishing behavior, and contributed to unethical behavior. Secondly the idea of publishing for high impact will be studied, as this is less well researched. Here the focus will not be on unethical behavior, but rather trends in publishing behavior will be examined to establish the link between a paradigmatic shift in what is important and publishing behavior. The period in which high impact publications became a criteria for evaluation will have to be identified, after which variables contributing to the impact of papers will be identified by means of a small literature study. These variables will be used to conduct a quantitative analysis of how these factors have changed over the years.

## Publish or Perish

Scholarly publication rates, Errami and Garner (2008) claim, are at an all-time high. This is not caused by an increase in productivity, but rather by changes in the way scholars publish (Broad 1981), which is linked to the pressure to publish (Errami and Garner 2008). A 1981 commentary in *Science* reported that co-authorship and multiple publication of the same data were on the rise, whilst the length of papers was decreasing (Broad 1981). The increase in co-authorship is attributable to interdisciplinary papers, multi-institutional clinical trials, but also to gratuitous listing of co-authors (Broad 1981). Gift authorship (Matías-Guiu and García-Ramos 2010), for instance including the head of a department or lab, is a common practice in some disciplines as is adding other researchers out of courtesy (Broad 1981), or an expectation of reciprocity (Webster et al. 2009).

In addition to a rise in co-authorship, a decrease in paper length was already noted in the early 1980s (Broad 1981). Scholars prefer to publish four short papers instead of one long paper (Broad 1981). They slice their data as if it were a salami, hence the term salami-slicing is sometimes used. The terms *Least Publishable Unit* (LPU) (Broad 1981), or *Smallest Publishable Unit* (SPU) (Elliott 2013) are used to describe these papers that contain the minimum amount of information needed to get published.

Another trend, closely related to salami slicing, is that of duplicate, or multiple, publication publishing articles that overlap substantially (Andreescu 2013; Kumar 2008; von Elm et al. 2004). This could be a simple copy (with the same authors, same data, basically same content, maybe a different title), a salami-sliced article without cross references to other articles based on the same data, a *meat extender* also called *data augmentation* (which is an expansion of an existing article with more data, sometimes without cross-reference), salami-sliced articles published by different authors (most common in multicenter trials), and a textual copy of an article with a different dataset and, possibly, different results and/or conclusion (Kumar 2008; von Elm et al. 2004).

Using text comparison software followed by manual verification, Errami and Garner (2008) uncovered a growing trend of duplicate publications in the biomedical literature; just below 2 per 1000 in 1975 to just below 10 in 1000 in 2005. Whilst this quintupled number still only represents 1 % of the papers, it is worrying since duplication represents just one possible mode of scientific misconduct.

The importance of having many publications is now declining in favor of the impact of publications (Franco 2013), although there are still scientists evaluated solely on the number of publications (Anderson et al. 2007). Impact is the focus of the next section.

## High Impact Publications

The idea that the importance of a publication can be judged from the number of references it receives is not recent. Even before Garfield (2006) first published about a Citation Index for Science, in 1955, the idea already existed, as he himself readily acknowledges. Early in the twentieth century, Gross and Gross (1927) postulated that the number of references a journal receives from a set of representative journals suggests something about its importance to the field, aiding librarians in choosing journals to add to their collections.

“The impact factor” states Moustafa, “became a major detrimental factor of quality, creating huge pressures on authors, editors, stakeholders and funders” (2014). But when did the impact of a single scholar, as measured by the citations (s)he receives, become important? This seems hard to pin point. In 1990, Tsafrir and Reis state “administrators are turning more to the citation performance of individuals” (1990) suggesting an increase in importance in or shortly prior to 1990, at least for Medicine. But it seems to have started earlier, in 1975 Wade provides cases where scholars’ citation counts were used for tenure and funding decisions, but it was by no means commonly used as an assessment tool at that time (Wade 1975). If indeed the idea about the importance of being cited influences scholars, consciously or unconsciously, we would expect this to be reflected in their work, starting between 1975 and 1990, in at least some scientific fields.

Recent research, discussed below, has examined the characteristics of papers, such as their writing style, which have an influence on subsequent citations. Whilst we should look at factors influencing subsequent citations in papers published in the period that we are interested into truly understand what was relevant then, the factors identified in current research offer some insights. These factors are expected to differ between the period before 1975 and the period after 1990, as the transition by then has already started.

The number of references a paper contains has been found to be positively correlated with the number of times a paper is cited, and this holds for all fields researched (Vieira and Gomes 2010; Webster et al. 2009; Wesel et al. 2014). Having many references can be useful to defend a paper against attacks (Latour 1987). Whilst references should be relevant to the paper, their numbers could be inflated by simply copying references from other papers (Ramos et al. 2012) or via a process of *I cite you, you cite me* in a form of reciprocal altruism (Webster et al. 2009).

The number of authors contributing to a paper is also a stable positive influencer across fields (Frenken et al. 2005; Glänzel and Thijs 2004; Levitt and Thelwall 2009; Vieira and Gomes 2010; Webster et al. 2009; Wesel et al. 2014). The rise of multi-authored papers, already observed by de Solla Price (1963) in the early 1960s, is often thought of as resulting from a rise in multi-disciplinary research. However other explanations for this rise are gratuitous listing of co-authors and gift-authorship, already mentioned above in the context of publish or perish. In the context of high-impact publication the naming of extra authors not only helps these authors gather extra publications, but could also help the paper to become highly cited, by extending the network to which the paper can easily be introduced (Frenken et al. 2005). Especially when eminent co-authors are named this has an even greater effect on the number of times a paper is cited (Haslam et al. 2008).

Another factor which, in most fields, correlates positively with the times an article is cited is its total length (Haslam et al. 2008; Hudson 2007; Vieira and Gomes 2010; Wang et al. 2012; Wesel et al. 2014), although this does not seem to hold in Applied Physics (Wesel et al. 2014). Notice, this seems to contrast with a trend observed for publish or perish which stimulates short, sliced, papers. Some suggests that lengthening is done to meet a presumed standard (Andreescu 2013).

Other interesting factors include the presence of a colon in the title and the length of the title (Haslam et al. 2008; Jacques and Sebire 2010). The direction of the effect of title length seems to differ across fields. In Sociology, Applied Physics, and a sub-set of PLoS journals a shorter title is associated with more citations (Jamali and Nikzad 2011; Wesel et al. 2014). Whilst in General and Internal Medicine the effect is reversed (Wesel et al. 2014).

The readability of abstracts also influences the number of citations an article receives, at least in Applied Physics and General and Internal Medicine (Wesel et al. 2014). A less readable than average abstract, as measured by the Flesch Reading Ease Score (Flesch 1948), has a positive effect on the number of citations an article receives. More sentences in the abstract is also related to more frequent citation in Sociology, Applied Physics, and General and Internal Medicine (Wesel et al. 2014).

The mechanism by which these factors are understood to influence the number of incoming citations is not relevant for this work (for exploration see, for instance, Wesel et al. 2014). What does matter is if the utilization of *tricks* that increase the number of received citations is increasing. These tricks do not necessarily represent scientific misconduct, although artificially inflating the author count, adding unnecessary references, and purposely making the abstract hard to read clearly can be considered misconduct. Depending on the circumstances this could also be said for lengthening a paper, if this lengthening occurs without adding new, relevant, information, this could be seen as misconduct.

Historically some of these, or related, factors have been shown to be stable whilst others are known to have changed. According to Gross et al. (2002) the number of citations per 100 words has risen from 0.3 in the period 1901–1925 to 1.8 in the period 1976–1995. This rise has been quite steep, in the period 1926–1950 there were 0.8 citations per 100 words and 1.5 in the period 1951–1975. The number of references quoted in articles was quite stable over a long period, in 1955 Garfield calculated an average of ten (Garfield 2006), and in the early ‘60s de Solla Price (1963) gives just under ten as the norm, stating it has been stable for many years.

## Reproducing of Practices

Scholars who have traits enabling them to produce more and higher cited papers than another scholar in the same field are more likely to secure resources, e.g. career, funding, PhD candidates and the like (Anderson et al. 2007). Since the relationship between a professor and a Ph.D. candidate is a socialization process, many Ph.D. candidates are influenced by the publishing style of their professors. Thus they pick up on traits about what constitutes good scholarly conduct and what constitutes misconduct. Furthermore, productive scholars will be read more, and are thus more likely to influence their readers with their style and approach to citation. Scholars, at all moments in their career but especially if they are new to the field, are further socialized by what they read, what they see, and what they hear from their peers and especially from those who are seen as successful.

Thus scholarly (mis)conduct is reproduced via a form of sociocultural evolution. The selection mechanism (Nolan and Lenski 2006) is evident, as described in the above paragraph. In other words; “selection theory takes the following from: when interactors interact, replicators create lineages by a process of selection” (Gross et al. 2002).

As such conducts becomes more widespread, scholars have come to see these practices as the norm, and as the accepted way to conduct science. As Elliott suggested when discussing salami-slicing “there is no intentional deceit taking place, just an assumption that this practice is perfectly acceptable” (2013).

## Expected Results

Following the discussion above one would expect to observe the following:

- A decrease in the length of the paper title, in most fields
- A rise in the number of authors contributing to a paper
- An increase in paper length
- An increase in the number of sentences in the abstract
- Most likely a decrease in readability of the abstract until it reaches an optimum
- A rise in the number of references a paper contains
- And an increase in paper titles with a (semi-)colon

Given the generational effect described above we would aspect these changes to accelerate, at least until reaching an optimum or plateau level.

## Methodology

To select representative journals, 50 journals with the highest Impact Factor for the years 1997 and 2012 from Thomson Reuters Journal Citation Reports (JCR) Science and Social Science edition were compared to identify journals which have been influential for many years.^2^ There was an overlap of 18 journals in the JCR Science Edition and 20 journals in the JCR Social Science Edition. For these journals the availability of data in Thomson Reuters Web of Knowledge was checked, as data was required from 1960 till 2004 in order to create three 15 year periods (1960–1974, 1975–1989, and 1990–2004) of which the first and third can be compared. Eight journals in the JCR Science Edition and four journals in the JCR Social Science Edition met this criterion.

Information about the papers which appeared in these journals was downloaded from the Web of Knowledge (WoK). WoK data provided information on the publication year, the title, the authors, the DOI. From the title, the length in the number of words, and presence of a (semi-)colon were recorded. From the list of authors, the number of authors was counted, by counting the separating semi-colons and adding 1, for papers with an anonymous author the author count field was left blank. Data on the number of references contained in the paper were also extracted from WoK, however this data was unavailable for papers published before 1988,^3^ and thus this variable was not analyzed. Using CrossRef^4^ the DOI was translated to the URL of the papers at the publisher’s website. When the DOI was missing, the article name, journal, and year were used to query CrossRef for the DOI, which was only accepted if the first author was listed and the match had a 100 % score. From the publishers website the abstract and type of paper were acquired, as well as the start and end page, as there was incongruity between publisher and WoK data. For *Chemical Reviews* and *Pharmacological Reviews* it proved not possible to obtain information about the paper type, thus these journals were removed from the sample.

HTML codes^5^ were removed from the abstract when necessary. Using the built-in readability function in Microsoft Word 2010 the Flesch Reading Ease was calculated. The formula used by Word (Microsoft 2007) for this is as follows;

$$\begin{aligned} {\text{Flesch}}\,{\text{Reading}}\,{\text{Ease}}\,{\text{Score}} & = 20 6. 8 3 5{-}\left( { 1.0 1 5\times {\text{Total}}\,{\text{Words}}/{\text{Total}}\,{\text{Sentences}}} \right) \\ &\quad {-}\left( { 8 4. 6\times {\text{Total}}\,{\text{Syllables}}/{\text{Total}}\,{\text{Words}}} \right) \end{aligned}$$

The Flesch Reading Ease Score (FRES) is a readability scale in which a higher score indicates easier readability, for all practical considerations the scale can be thought of as ranging from 0 to 100, where a score from 0 to 30 indicates very difficult and a score from 90 to 100 very easy.

Three rough categories of paper types were deemed suitable for analysis; Articles (review and original), Letters, and short scientific communications. This leads to the fifteen journal paper type combinations shown in Table 1. Differences in naming had to be resolved, for instance *Correspondence* and *Letters to the Editor* in *Lancet* and in *Nature* were combined for their respective journals.

**Table 1:** Number of papers in the dataset

	Total	1960–1974	1975–1989	1990–2004
American Psychologist; Comment and Reply JCR SSE: Psychology, multidisciplinary	1099	92	181	826
American Psychologist; Journal Article JCR SSE: Psychology, multidisciplinary	3144	863	868	1413
Annual Review of Biochemistry; review-article JCR SE: Biochemistry and molecular biology	952	351	321	280
Annual Review of Physiology; review-article JCR SE: Physiology	890	258	333	299
Annual Review of Psychology; review-article^a^ JCR SSE: Psychology, multidisciplinary	517	162	116	239
Lancet; article^b^ JCR SE: Medicine, general and internal	6213	3777	0	2436
Lancet; hypothesis^c^ JCR SE: Medicine, general and internal	690	256	319	115
Lancet; letters to the editor JCR SE: Medicine, general and internal	55,703	10,852	22,421	22,430
Nature; article JCR SE: Multidisciplinary sciences	7522	4859	1481	1182
Nature; letters to editor and correspondence JCR SE: Multidisciplinary sciences	50,450	18,812	13,389	18,249
Physiological Reviews; article JCR SE: Physiology	1019	248	311	460
Psychological Bulletin; journal article JCR SSE: Psychology, multidisciplinary	1795	620	547	628
Psychological Review; journal article JCR SSE: Psychology, multidisciplinary	1021	392	174	455
Science; letters^d^ JCR SE: Multidisciplinary sciences	3070	1400	752	918
Science; report JCR SE: Multidisciplinary sciences	32,475	11,857	9882	10,736
Total	166,560	54,799	51,095	60,666

^a^Some years with zero papers, most likely due to a low number of papers overall

^b^For the period 1972–1990 there were no papers identified as article, this is due to how Science Direct displayed paper identification for part of the papers

^c^Some years with zero papers, are most likely due to a low number of papers overall

^d^One paper in 1997 and zero in 1998–1999, reason unknown

These variables were compared using an independent-samples *t*-test grouping the papers in the period 1960–1974 and 1990–2004. The presence of a (semi-)colon in the title was compared using the Chi square test. Effect size was calculated using the *r* = sqrt((t^2^/(t^2^ + *df*)) formula for the *t* test, resulting in only positive effect sizes. And ϕ = sqrt(*x* ^2^/n) for the Chi square test, also resulted in only positive effect sizes.

## Results

Paper titles, measured by the number of words, are longer in the second period, 1990–2004, for fourteen of the fifteen sets, only *Letters to editor* and *correspondence* in *Nature* show a reduction in title length (see Table 8). An independent-samples *T* test determines these differences are significant (*p* < 0.05), even for this before mentioned outlier (Table 2). The effect sizes for the different sets vary from small to large, the effect size is smallest for *Nature; letters to the editor* and *correspondence* and largest for *Annual Review of Psychology; review*-*article* (Table 8). Fourteen out of fifteen sets behave contrary to the prediction.

**Table 2:** *T* test statistics for title length

	*t*	Degrees of freedom	*p* value
American Psychologist; Comment and Reply	−4.091	916	.000
American Psychologist; Journal Article	−7.908	1934.237	.000
Annual Review of Biochemistry; review-article	−10.968	488.989	.000
Annual Review of Physiology; review-article	−14.677	554.791	.000
Annual Review of Psychology; review-article	−14.135	390.756	.000
Lancet; article	−41.234	4311.862	.000
Lancet; hypothesis	−4.751	369	.000
Lancet; letters to the editor	−32.026	24,949.985	.000
Nature; article	−11.622	2130.690	.000
Nature; letters to editor and correspondence	18.068	37,059	.000
Physiological Reviews; article	−7.323	706	.000
Psychological Bulletin; journal article	−12.287	1214.666	.000
Psychological Review; journal article	−7.120	844.862	.000
Science; letters	−8.300	1653.707	.000
Science; report	−31.809	22,535.249	.000

For all fifteen sets the mean number of authors per paper increases between the two periods (Table 9) and these increases are significant for all sets (Table 3). The effect sizes for the different sets vary from halfway between small and medium to large, the effect size is smallest for *American Psychologist; Comment* and *Reply* and largest for *Science; report* (see Table 9). All fifteen sets follow the predicted behavior.

**Table 3:** *T* test statistics for author count

	*t*	Degrees of freedom	*p* value
American Psychologist; Comment and Reply	−2.025	110.103	.045
American Psychologist; Journal Article	−11.139	1559.752	.000
Annual Review of Biochemistry; review-article	−7.776	420.278	.000
Annual Review of Physiology; review-article	−7.293	475.893	.000
Annual Review of Psychology; review-article	−5.945	399	.000
Lancet; article	−14.719	2459.036	.000
Lancet; hypothesis	−6.417	161.699	.000
Lancet; letters to the editor	−37.279	29,862.353	.000
Nature; article	−11.469	1154.539	.000
Nature; letters to editor and correspondence	−48.675	18,690.428	.000
Physiological Reviews; article	−12.311	667.857	.000
Psychological Bulletin; journal article	−14.569	1079.846	.000
Psychological Review; journal article	−12.698	713.441	.000
Science; letters	−7.398	1254.141	.000
Science; report	−70.026	12,685.621	.000

The page count increases for ten out of the fifteen sets, in the other five the page count decreases (Table 10). The changes are significant in twelve sets, for *American Psychologist; Comment* and *Reply,* *American Psychologist; Journal Article,* and *Lancet; hypothesis* the change is not significant at all (Table 4). The effect sizes for the sets in which the change is significant vary from very small for *Lancet; letters to the editor* to very large for *Science; report* (see Table 10). Most sets behave as predicted.

**Table 4:** *T* test statistics for page count

	*t*	Degrees of freedom	*p* value
American Psychologist; Comment and Reply	.119	100.707	.905
American Psychologist; Journal Article	−1.174	1266.782	.241
Annual Review of Biochemistry; review-article	−2.585	626	.010
Annual Review of Physiology; review-article	8.671	437.489	.000
Annual Review of Psychology; review-article	5.054	213.284	.000
Lancet; article	−47.424	4245.280	.000
Lancet; hypothesis	−.163	320.211	.871
Lancet; letters to the editor	−6.702	26,140.010	.000
Nature; article	−40.168	6039	.000
Nature; letters to editor and correspondence	−84.856	25,727.743	.000
Physiological Reviews; article	4.149	380.383	.000
Psychological Bulletin; journal article	−13.658	1194.349	.000
Psychological Review; journal article	−15.381	815.745	.000
Science; letters	3.144	2163.076	.002
Science; report	−112.927	22,494.270	.000

The number of sentences in the abstract rises in seven of the nine sets for which statistics for the number of sentences in the abstract could be calculated, for the other two, from the same journal, this number decreased (Table 11). These differences are significant (Table 5). The effect sizes for the different sets vary from small to very large the effect size is smallest for *American Psychologist; Journal Article* and largest for *Lancet; article* and *Nature; Article* (Table 11). This rise is in line with the predicted behavior.

**Table 5:** *T* test statistics for number of sentences in the abstract

	*t*	Degrees of freedom	*p* value
American Psychologist; Comment and Reply	3.745	104.597	.000
American Psychologist; Journal Article	2.290	1336.504	.022
Lancet; article	−72.459	3308.154	.000
Lancet; hypothesis	−5.853	283	.000
Nature; article	−48.565	1497.515	.000
Nature; letters to editor and correspondence	−120.598	30,682.537	.000
Psychological Bulletin; journal article	−6.858	1243	.000
Psychological Review; journal article	−3.198	762.092	.001
Science; report	−97.789	21,751.445	.000

In eight out of nine sets examined, the Flesch Reading Ease Score of the abstract is lower in the period 1990–2004 then it was in the period 1960–1974 (Table 12), these differences are significant (Table 6). This suggests that for eight of these sets the abstracts became harder to read, only *Lancet; Articles* became easier to read. The effect sizes for the different sets vary from small to halfway between small and medium, the effect size is smallest for *Lancet; article* and *Nature; letters to editor* and *correspondence* and largest for *American Psychologist; Comment* and *Reply* (see Table 12). As predicted, abstracts became harder to read.

**Table 6:** *T* test statistics for the abstracts Flesch Reading Ease score

	*t*	Degrees of freedom	*p* value
American Psychologist; Comment and Reply	5.149	104.597	.000
American Psychologist; Journal Article	10.257	1445.817	.000
Lancet; article	−4.796	2124.720	.000
Lancet; hypothesis	2.656	260.642	.008
Nature; article	13.584	2854.784	.000
Nature; letters to editor and correspondence	13.915	30,210.639	.000
Psychological Bulletin; journal article	7.309	1224.682	.000
Psychological Review; journal article	6.339	842	.000
Science; report	20.396	22,096.376	.000

For five of the fifteen sets the proportion of titles with a (semi-)colon in the title rises, for two the proportion stays about the same, and for seven the proportion is lower when we compare the period 1960–1974 to 1990–2004 (see Tables 13, 14, 15, 16, 17, 18, 19, 20, 21, 22, 23, 24, 25, 26, 27). And these changes were found to be significant for the twelve sets in which we see a rise or drop (Table 7). The effect sizes for the sets in which the change is significant vary from small to halfway between medium and large, the effect size is smallest for *Nature; article* and largest for *Lancet; article* (Table 7). For some of the sets the predicted behavior is followed, others follow the opposite behavior.

**Table 7:** Chi square statistics for colon in title

	Value	Degrees of freedom	*p* value	ϕ
American Psychologist; Comment and Reply	29.187	1	.000	.18
American Psychologist; Journal Article	261.596	1	.000	.34
Annual Review of Biochemistry; review-article	35.716	1	.000	.24
Annual Review of Physiology; review-article	.018	1	.892	.01
Annual Review of Psychology; review-article	42.514	1	.000	.33
Lancet; article	1191.184	1	.000	.44
Lancet; hypothesis	.487	1	.485	.04
Lancet; letters to the editor	2400.234	1	.000	.27
Nature; article	73.528	1	.000	.11
Nature; letters to editor and correspondence	562.801	1	.000	.12
Physiological Reviews; article	33.286	1	.000	.22
Psychological Bulletin; journal article	.001	1	.980	.00
Psychological Review; journal article	12.218	1	.000	.12
Science; letters	22.192	1	.000	.10
Science; report	2830.663	1	.000	.35

**Fig. 1:**
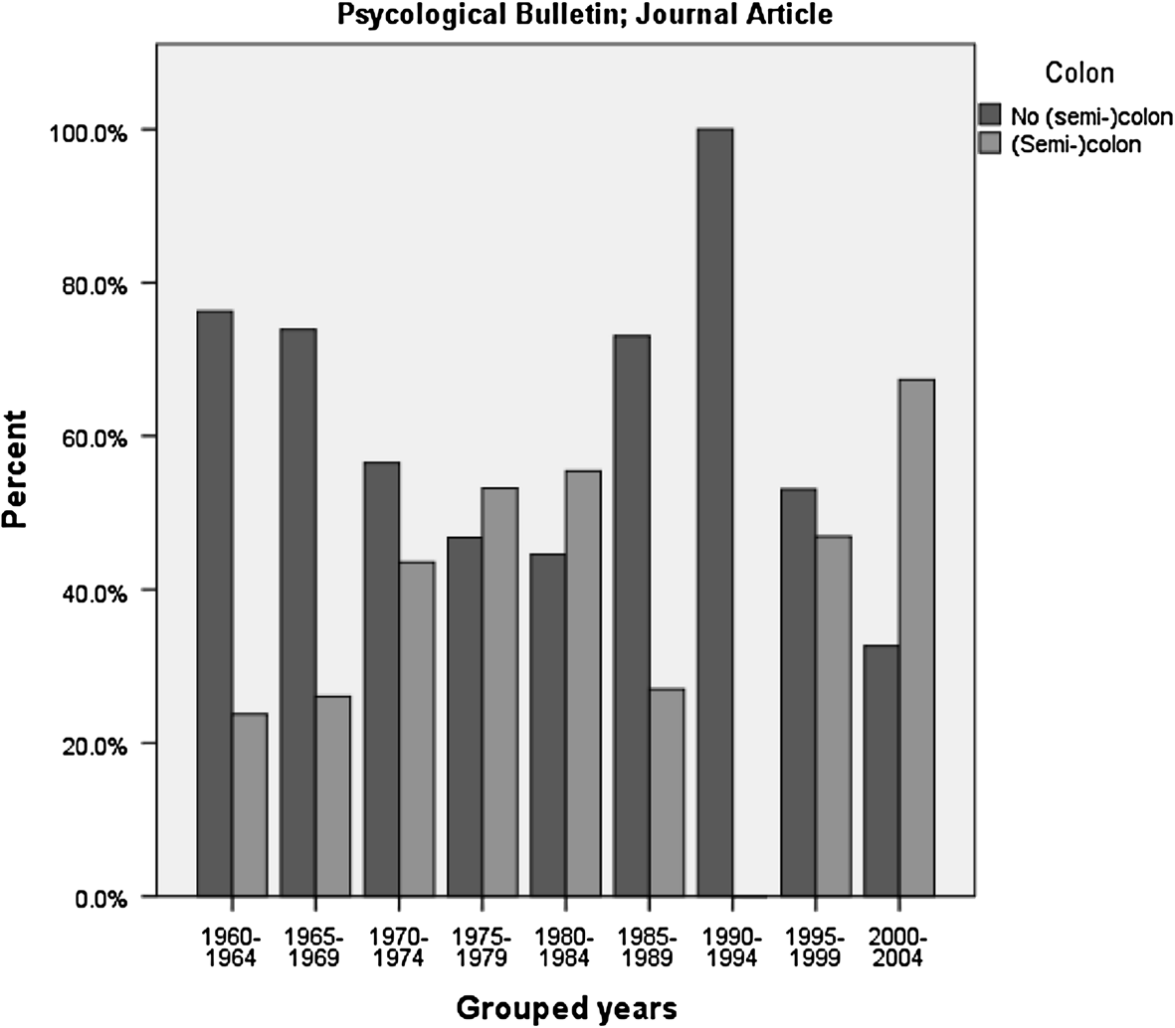
Use of colon in title for Psychological Bulletin; journal article

**Fig. 2:**
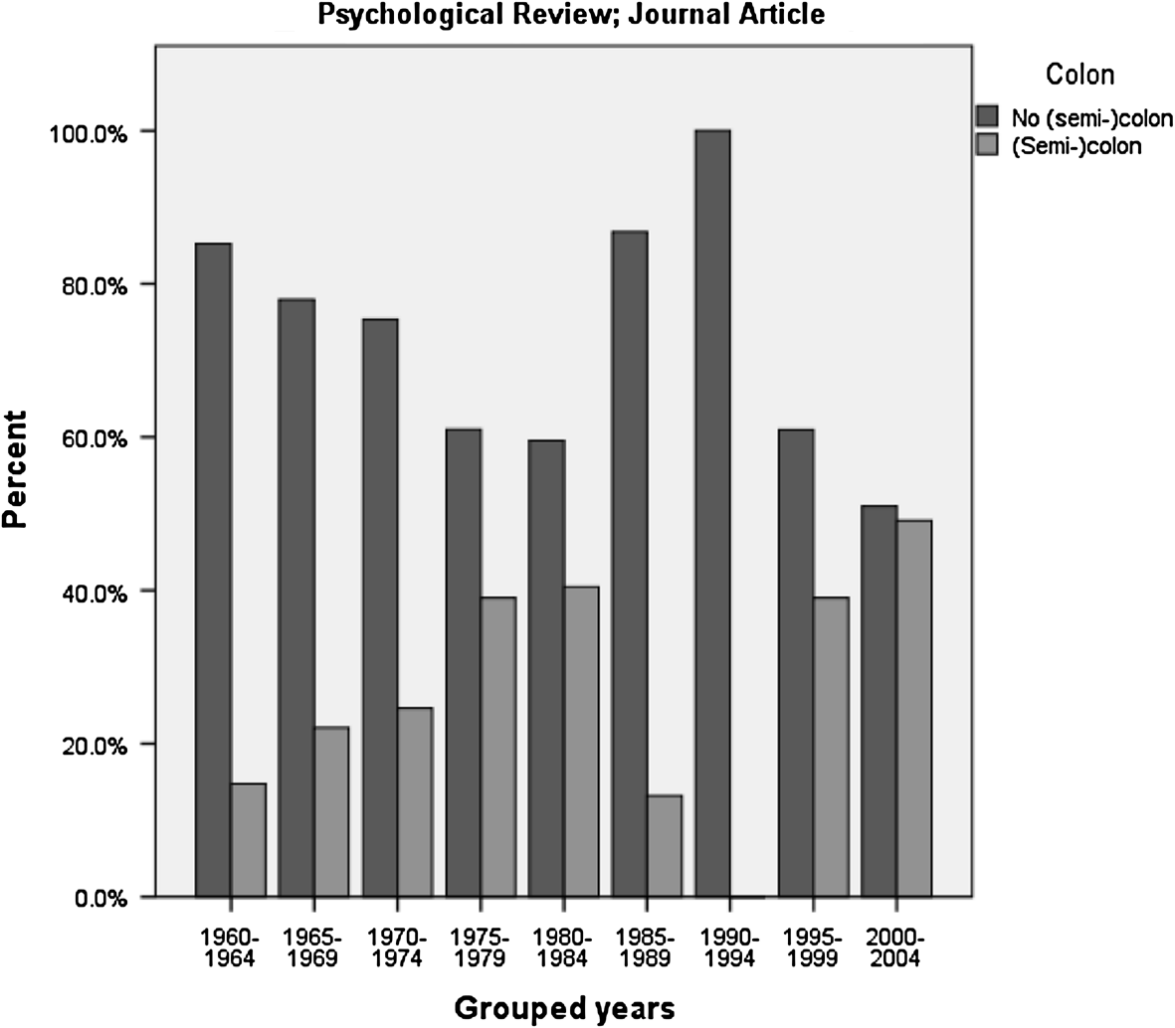
Use of colon in title for Psychological Review; journal article

This observation might, however, be misguided, as it appears that in the period 1990–1994, and most likely some surrounding years, the number of (semi-)colon in titles was very low, which might point to a discrepancy in the way Web of Knowledge treated this character (see the exemplary graphs in Figs. 1, 2). For instance the articles noted on APA PsycNET as “Support theory: A nonextensional representation of subjective probability” and “Simultaneous over- and underconfidence: The role of error in judgment processes.” are registered in WOK as “Support theory—A nonextensional representation of subjective probability” and “Simultaneous over- and underconfidence—The role of error in judgment processes” respectively. Note this is not only limited to these two journals, but occurs throughout the dataset for this period.

## Conclusion and Discussion

Whilst the predicted pattern is followed in most cases there are notable exceptions such as the title length, for which the predicted pattern is only followed in one set. For other variables the predicted pattern is followed more closely, for every set the number of authors increases, although the single-authored paper did not die out, something de Solla Price (1963) predicted would happen for *Chemical Abstracts*, not included in this sample, by 1980. For the other cases there are one or more sets not following the predicted behavior. Exception being the presence of a colon with in the title, which only rises in six sets, but might have an alternative explanation (see “Results” section).

Given only fifteen journal/paper type sets, representing ten journals, which in turn represent four JCR Science Edition categories and only one JCR Social Science Edition, were included in this paper some explanation for changes in individual variables can be sought in changes in the journal’s editorial policies or in changes in the field. A change in policies could have caused the change in the number of authors, but this would then have to have happened to for all journals examined. External factors of influence, other than the ideas that citing for impact and publish or perish are important, could also explain changes. The decline in readability could be caused by the rise in the use of word-processing software. Tin and Inggeris (2000) did find that students produce more complex text when word processing than when writing with pen and paper. Changes in the field could both be caused by changes related and not related to evaluation criteria based on publish or perish or on publishing for impact.

The rise in authors could be explained by a rise in multidisciplinary research. However this has already been dismissed as the sole explanation in earlier writings (Broad 1981; Matías-Guiu and García-Ramos 2010). Maybe the extravagant number of authors listed on some articles is not the result of an attempt to beat the Publish or Perish game or an attempt to become highly cited. Maybe it is a genuine attempt to acknowledge the contribution of those without whose work the research would not have been possible, such as lab technicians, doctors collecting data in their practices, and technicians keeping the machines of big science up and running. Instrumental and valuable, but not authors. Their contributions are perhaps too great to merely thank them in the Acknowledgements. Thus perhaps one solution is to find an alternative form of recognition that fills this apparent void between ‘Acknowledgement’ and ‘author’ ought to be filled. Gratuitous listing of co-authors greatly devalues authorship, something overlooked when research performance is evaluated. With authorship comes rights, the right for individuals to put a publication in their CVs, the right for a department or institution to claim the output. But authorship also comes with responsibility, as all authors are responsible for the content, right or wrong (for the latter, including responsibility for fraudulent actions, such as plagiarism and data fraud). Is the 50th author willing to bear this responsibility, responsibility for an article (s)he did not witness being created and might not even have read before it was submitted for publication?

Given the predicted behavior is followed in most of the cases, and there is a realistic case for why these changes occurred, it is not unreasonable to link the observed changes in publication behavior to a change in evaluation criteria, which is also not out-of-line with what commonsense would predict. These findings combined with those of other researchers, for instance the link between high impact publications and hot topics (Moustafa 2014), lead to the conclusion that evaluation criteria act as switchmen, determining the tracks along which scholarly work is pushed by the dynamic of interests of both scholars and their institutions.

Both the changes which follow as well as those which are counter to the expected behavior could be explained by the journal’s editorial policies. It would be possible to discover such policies by studying editorials and comments on submitted manuscripts. This would establish if editorial policies could have influenced these variables, but will, most likely, not explain what caused the change in policies, which could also be a response to external evaluation criteria.

We also need to consider the underlying cause of the rise of these ideas: why have they become so dominant in science? This is a harder question to answer, and one needs to work through national and university policy documents in order to find an answer to this question. One explanation might be sought in changes in how governments try to justify expenditures. Starting in the early 1980s university policies have increasingly been influenced by a need for accountability, at least in EU countries (Geuna 2001). Without a Citation Index it is questionable if the number of citations would be as important as it is now. This is not a technologically deterministic stance (Smith and Marx 1994), a feasible way of counting citations was needed to facilitate the operationalization of the idea, or in other words “knowledge is embedded in and performed by infrastructures” (Wyatt et al. 2013). Most likely the negative effect of the competition system for distributing careers, funding and the like on the behavior of scientist has been underestimated by those who bestow the rewards (Anderson et al. 2007).

An interesting future research opportunity would be to compare how these trends differ between fields. Some are traditionally more book- than article-based, notably in the humanities, and still try to hold on to these traditions. Such publications are less easily evaluated by criteria which are predominantly used in the STEM and medical fields.

This research has aimed to find whether publications and citation pressures have resulted in changes in the number of papers scholars produce and in their characteristics, but there are other, potentially more fruitful approaches. Another method, more in line with Weber, would be to examine the *teachings* of practices, or to study the *theology* 6 of the ideas (for inspiration Weber 1958). These *teachings* could be distilled from scholarly guidebooks, such as methods texts, editorial guidelines or codes of practice, by looking for suggestions about salami slicing, duplicate publications, multi-authorship, inflating references, etc. The *theology* could be found in policies on promotion (such as the granting of tenure), basis for research funding, and the basis for rankings on university and (inter)national levels.

## Appendix

See Tables 8, 9, 10, 11, 12, 13, 14, 15, 16, 17, 18, 19, 20, 21, 22, 23, 24, 25, 26 and 27.

**Table 8:** Statistics for title length

	N	Mean	SD	Minimal	Maximal	*r*
American Psychologist; Comment and Reply						
1960–1974	92	5.32	2.94	1.00	19.00	.13
1990–2004	826	6.65	2.96	1.00	24.00	
American Psychologist; Journal Article						
1960–1974	863	7.95	3.93	1.00	36.00	.18
1990–2004	1413	9.34	4.26	1.00	33.00	
Annual Review of Biochemistry; review-article						
1960–1974	351	4.23	2.39	1.00	14.00	.44
1990–2004	280	6.83	3.34	1.00	21.00	
Annual Review of Physiology; review-article						
1960–1974	258	4.16	2.67	1.00	14.00	.53
1990–2004	299	7.78	3.16	1.00	20.00	
Annual Review of Psychology; review-article						
1960–1974	162	2.70	1.96	1.00	15.00	.58
1990–2004	239	6.47	3.37	1.00	18.00	
Lancet; article						
1960–1974	3777	8.34	3.60	1.00	33.00	.53
1990–2004	2436	12.87	4.59	2.00	34.00	
Lancet; hypothesis						
1960–1974	256	7.61	3.46	1.00	21.00	.24
1990–2004	115	9.52	3.83	2.00	24.00	
Lancet; letters to the editor						
1960–1974	10,852	5.12	2.21	1.00	21.00	.20
1990–2004	22,430	6.00	2.61	1.00	22.00	
Nature; article						
1960–1974	4859	9.03	4.02	1.00	28.00	.24
1990–2004	1182	10.33	3.28	2.00	23.00	
Nature; letters to editor and correspondence						
1960–1974	18,812	9.13	3.74	1.00	36.00	.09
1990–2004	18,249	8.44	3.62	1.00	34.00	
Physiological Reviews; article						
1960–1974	248	6.13	2.93	1.00	15.00	.27
1990–2004	460	7.90	3.16	1.00	18.00	
Psychological Bulletin; journal article						
1960–1974	620	7.83	3.34	1.00	22.00	.33
1990–2004	628	10.38	3.98	2.00	24.00	
Psychological Review; journal article						
1960–1974	392	7.85	3.39	1.00	25.00	.24
1990–2004	455	9.65	3.99	2.00	26.00	
Science; letters						
1960–1974	1400	3.76	1.53	1.00	14.00	.20
1990–2004	918	4.39	1.92	1.00	19.00	
Science; report						
1960–1974	11,857	8.87	2.96	1.00	58.00	.21
1990–2004	10,736	10.09	2.82	1.00	23.00

**Table 9:** Statistics for author count

	N	Mean	SD	Minimal	Maximal^a^	*R*
American Psychologist; Comment and Reply						
1960–1974	89	1.24	.87	1.00	7.00	.19
1990–2004	825	1.43	.91	1.00	11.00	
American Psychologist; Journal Article						
1960–1974	758	1.25	.62	1.00	6.00	.27
1990–2004	1111	1.82	1.55	1.00	20.00	
Annual Review of Biochemistry; review-article						
1960–1974	351	1.54	.67	1.00	5.00	.35
1990–2004	280	2.15	1.17	1.00	9.00	
Annual Review of Physiology; review-article						
1960–1974	258	1.41	.62	1.00	4.00	.32
1990–2004	299	1.97	1.13	1.00	12.00	
Annual Review of Psychology; review-article						
1960–1974	162	1.46	.64	1.00	4.00	.29
1990–2004	239	1.95	.90	1.00	6.00	
Lancet; article						
1960–1974	3777	2.69	1.49	1.00	12.00	.28
1990–2004	2436	7.80	17.07	1.00	697.00^b^	
Lancet; hypothesis						
1960–1974	256	1.69	1.00	1.00	8.00	.45
1990–2004	115	2.67	1.49	1.00	8.00	
Lancet; letters to the editor						
1960–1974	10,852	1.90	1.21	1.00	19.00	.21
1990–2004	22,430	2.52	1.79	1.00	39.00	
Nature; article						
1960–1974	4622	2.24	1.34	1.00	16.00	.32
1990–2004	1153	9.07	20.20	1.00	241.00^c^	
Nature; letters to editor and correspondence						
1960–1974	17,853	2.15	1.12	1.00	13.00	.34
1990–2004	17,132	4.11	5.14	1.00	349.00^d^	
Physiological Reviews; article						
1960–1974	248	1.40	.57	1.00	3.00	.43
1990–2004	460	2.32	1.39	1.00	10.00	
Psychological Bulletin; journal article						
1960–1974	620	1.36	.69	1.00	6.00	.41
1990–2004	628	2.10	1.06	1.00	6.00	
Psychological Review; journal article						
1960–1974	392	1.36	.62	1.00	5.00	.43
1990–2004	455	2.16	1.16	1.00	9.00	
Science; letters						
1960–1974	1400	1.35	1.08	1.00	16.00	.20
1990–2004	918	1.89	2.04	1.00	29.00	
Science; report						
1960–1974	11,857	2.37	1.34	1.00	31.00	.53
1990–2004	10,736	5.35	4.22	1.00	93.00	

^a^In the whole sample of 166,560 papers only 27 papers have 100 or more authors, and 67 have 50 or more

^b^Ledergerber and PLATO Collaboration (2004)

^c^Gibbs et al. (2004)

^d^Abazov et al. (2004)

**Table 10:** Statistics for page count

	N	Mean	SD	Minimal	Maximal	*r*
American Psychologist; Comment and Reply						
1960–1974	92	2.23	1.58	1.00	13.00	.01
1990–2004	692	2.21	.99	1.00	13.00	
American Psychologist; Journal Article						
1960–1974	863	7.72	11.87	1.00	166.00	.03
1990–2004	1406	8.25	7.30	1.00	176.00	
Annual Review of Biochemistry; review-article						
1960–1974	351	31.32	10.63	8.00	73.00	.10
1990–2004	279	33.45	9.73	9.00	71.00	
Annual Review of Physiology; review-article						
1960–1974	258	31.88	11.67	10.00	84.00	.38
1990–2004	299	24.46	7.81	2.00	56.00	
Annual Review of Psychology; review-article						
1960–1974	162	33.45	10.59	12.00	92.00	.33
1990–2004	239	28.92	5.16	18.00	57.00	
Lancet; article						
1960–1974	3777	3.44	1.22	1.00	12.00	.59
1990–2004	2435	5.24	1.59	1.00	14.00	
Lancet; hypothesis						
1960–1974	256	3.11	1.14	1.00	9.00	.01
1990–2004	115	3.12	.75	1.00	5.00	
Lancet; letters to the editor						
1960–1974	10,828	1.38	.49	1.00	4.00	.04
1990–2004	22,413	1.49	2.37	1.00	346.00	
Nature; article						
1960–1974	4859	3.44	2.50	1.00	93.00	.46
1990–2004	1182	6.51	1.61	1.00	29.00	
Nature; letters to editor and correspondence						
1960–1974	18,812	2.01	.71	1.00	34.00	.47
1990–2004	18,249	3.06	1.52	1.00	52.00	
Physiological Reviews; article						
1960–1974	248	49.08	29.37	7.00	321.00	.21
1990–2004	460	40.38	20.55	1.00	194.00	
Psychological Bulletin; journal article						
1960–1974	620	13.97	7.43	1.00	51.00	.37
1990–2004	627	20.46	9.26	1.00	70.00	
Psychological Review; journal article						
1960–1974	392	13.98	7.36	2.00	52.00	.47
1990–2004	454	23.38	10.33	3.00	63.00	
Science; letters						
1960–1974	1400	3.10	6.06	1.00	105.00	.07
1990–2004	918	2.36	5.17	1.00	112.00	
Science; report						
1960–1974	11,857	2.60	.86	1.00	23.00	.60
1990–2004	10,736	3.87	.83	1.00	20.00

**Table 11:** Statistics for number of sentences in abstract

	N	Mean	SD	Minimal	Maximal	*r*
American Psychologist; Comment and Reply						
1960–1974	92	4.02	2.72	.00	16.00	.34
1990–2004	793	2.94	1.33	.00	9.00	
American Psychologist; Journal Article						
1960–1974	815	4.38	2.28	.00	17.00	.06
1990–2004	1405	4.17	1.69	.00	11.00	
Annual Review of Biochemistry; review-article						
1960–1974	1	11.00	–	11.00	11.00	
1990–2004	233	5.87	2.50	1.00	17.00	
Annual Review of Physiology; review-article						
1960–1974	2	8.00	7.07	3.00	13.00	
1990–2004	253	5.80	2.67	1.00	16.00	
Annual Review of Psychology; review-article						
1960–1974	0	–	–	–	–	
1990–2004	210	4.73	1.95	.00	11.00	
Lancet; article						
1960–1974	1264	4.70	2.23	.00	19.00	.78
1990–2004	2424	11.12	3.07	1.00	25.00	
Lancet; hypothesis						
1960–1974	181	4.07	2.35	.00	11.00	.33
1990–2004	104	5.72	2.21	1.00	13.00	
Lancet; letters to the editor						
1960–1974	0	–	–	–	–	
1990–2004	10,370	2.70	1.64	.00	17.00	
Nature; article						
1960–1974	1683	1.86	.84	.00	8.00	.78
1990–2004	1179	4.74	1.91	1.00	13.00	
Nature; letters to editor and correspondence						
1960–1974	18,288	4.11	2.29	.00	36.00	.57
1990–2004	12,865	6.93	1.82	.00	26.00	
Physiological Reviews; article						
1960–1974	0	–	–	–	–	
1990–2004	338	9.96	3.94	2.00	26.00	
Psychological Bulletin; journal article						
1960–1974	617	4.61	1.79	.00	11.00	.19
1990–2004	628	5.29	1.69	.00	11.00	
Psychological Review; journal article						
1960–1974	389	5.06	2.05	.00	16.00	.12
1990–2004	455	5.49	1.73	.00	14.00	
Science; letters						
1960–1974	4	3.00	.82	2.00	4.00	
1990–2004	6	2.67	1.21	2.00	5.00	
Science; report						
1960–1974	11,701	3.05	1.28	.00	18.00	.55
1990–2004	10,724	4.80	1.40	.00	21.00

**Table 12:** Statistics for Flesch Reading Ease Score for the abstract

	N	Mean	SD	Minimal	Maximal	*r*
American Psychologist; Comment and Reply						
1960–1974	92	18.85	14.32	.00	65.70	.45
1990–2004	793	10.88	11.32	.00	53.20	
American Psychologist; Journal Article						
1960–1974	815	20.46	14.68	.00	100.00	.26
1990–2004	1405	14.24	12.04	.00	67.20	
Annual Review of Biochemistry; review-article						
1960–1974	1	34.80	–	34.80	34.80	
1990–2004	233	15.16	13.06	.00	55.90	
Annual Review of Physiology; review-article						
1960–1974	2	42.40	2.55	40.60	44.20	
1990–2004	253	13.42	11.44	.00	54.70	
Annual Review of Psychology; review-article						
1960–1974	0	–	–	–	–	
1990–2004	210	13.29	11.17	.00	42.20	
Lancet; article						
1960–1974	1264	20.01	13.55	.00	60.50	.10
1990–2004	2424	22.12	10.85	.00	53.60	
Lancet; hypothesis						
1960–1974	181	14.21	12.61	.00	49.70	.16
1990–2004	104	10.67	9.66	.00	38.80	
Lancet; letters to the editor						
1960–1974	0	–	–	–	–	
1990–2004	10,370	22.01	16.13	.00	87.40	
Nature; article						
1960–1974	1683	26.29	16.91	.00	80.70	.25
1990–2004	1179	18.85	12.37	.00	67.60	
Nature; letters to editor and correspondence						
1960–1974	18,288	20.66	13.89	.00	80.80	.08
1990–2004	12,865	18.64	11.65	.00	90.00	
Physiological Reviews; article						
1960–1974	0	–	–	–	–	
1990–2004	338	13.88	10.78	.00	48.70	
Psychological Bulletin; journal article						
1960–1974	617	18.57	12.88	.00	57.50	.20
1990–2004	628	13.49	11.60	.00	50.30	
Psychological Review; journal article						
1960–1974	389	20.71	13.05	.00	62.10	.21
1990–2004	455	15.25	11.95	.00	53.20	
Science; letters						
1960–1974	4	19.30	5.38	15.10	26.60	
1990–2004	6	27.53	15.41	9.80	53.10	
Science; report						
1960–1974	11,701	19.54	14.89	.00	81.30	.14
1990–2004	10,724	15.86	12.07	.00	74.20

**Table 13:** Crosstabulation Period* Colon in title American Psychologist; Comment and reply

	Colon in title		Total
	No	Yes	
Period			
1960–1974	67 (72.8 %)	25 (27.2 %)	92
1990–2004	753 (91.2 %)	73 (8.8 %)	826
Total	820	98

**Table 14:** Crosstabulation Period* Colon in title American Psychologist; Journal article

	Colon in title		Total
	No	Yes	
Period			
1960–1974	635 (73.6 %)	228 (26.4 %)	863
1990–2004	1363 (96.5 %)	50 (3.5 %)	1413
Total	1998	278

**Table 15:** Crosstabulation Period* Colon in title Annual Review of Biochemistry; review-article

	Colon in title		Total
	No	Yes	
Period			
1960–1974	333 (94.9 %)	18 (5.1 %)	351
1990–2004	222 (79.3 %)	58 (20.7 %)	280
Total	555	76	631

**Table 16:** Crosstabulation Period* Colon in title Annual Review of Physiology; review-article

	Colon in title		Total
	No	Yes	
Period			
1960–1974	198 (76.7 %)	60 (23.3 %)	258
1990–2004	228 (76.3 %)	71 (23.7 %)	299
Total	426	131

**Table 17:** Crosstabulation Period* Colon in title Annual Review of Psychology; review-article

	Colon in title		Total
	No	Yes	
Period			
1960–1974	155 (95.7 %)	7 (4.3 %)	162
1990–2004	165 (69.0 %)	74 (31.0 %)	239
Total	320	81

**Table 18:** Crosstabulation Period* Colon in title Lancet; article

	Colon in title		Total
	No	Yes	
Period			
1960–1974	3669 (97.1 %)	108 (2.9 %)	3777
1990–2004	1573 (64.6 %)	863 (35.4 %)	2436
Total	5242	971

**Table 19:** Crosstabulation Period* Colon in title Lancet; hypothesis

	Colon in title		Total
	No	Yes	
Period			
1960–1974	233 (91.0 %)	23 (9.0 %)	256
1990–2004	102 (88.7 %)	13 (11.3 %)	115
Total	335	36

**Table 20:** Crosstabulation Period* Colon in title Lancet; letters to the editor

	Colon in title		Total
	No	Yes	
Period			
1960–1974	8474 (78.1 %)	2378 (21.9 %)	10,852
1990–2004	21,407 (95.4 %)	1023 (4.6 %)	22,430
Total	29,881	3401

**Table 21:** Crosstabulation Period* Colon in title Nature; article

	Colon in title		Total
	No	Yes	
Period			
1960–1974	4468 (92.0 %)	391 (8.0 %)	4859
1990–2004	1169 (98.9 %)	13 (1.1 %)	1182
Total	5637	404

**Table 22:** Crosstabulation Period* Colon in title Nature; Letters to editor and correspondence

	Colon in title		Total
	No	Yes	
Period			
1960–1974	17,748 (94.3 %)	1064 (5.7 %)	18,812
1990–2004	18,037 (98.8 %)	212 (1.2 %)	18,249
Total	35,785	1276

**Table 23:** Crosstabulation Period* Colon in title Physiological Reviews; article

	Colon in title		Total
	No	Yes	
Period			
1960–1974	237 (95.6 %)	11 (4.4 %)	248
1990–2004	365 (79.3 %)	95 (20.7 %)	460
Total	602	106

**Table 24:** Crosstabulation Period* Colon in title Psychological Bulletin; journal article

	Colon in title		Total
	No	Yes	
Period			
1960–1974	434 (70.0 %)	186 (30.0 %)	620
1990–2004	440 (70.1 %)	188 (29.9 %)	628
Total	874	374

**Table 25:** Crosstabulation Period* Colon in title Psychological Review; Journal Article

	Colon in title		Total
	No	Yes	
Period			
1960–1974	315 (80.4 %)	77 (19.6 %)	392
1990–2004	318 (69.9 %)	137 (30.1 %)	455
Total	633	214

**Table 26:** Crosstabulation Period* Colon in title Science; letters

	Colon in title		Total
	No	Yes	
Period			
1960–1974	1265 (90.4 %)	135 (9.6 %)	1400
1990–2004	878 (95.6 %)	40 (4.4 %)	918
Total	2143	175

**Table 27:** Crosstabulation Period* Colon in title Science; report

	Colon in title		Total
	No	Yes	
Period			
1960–1974	7431 (62.7 %)	4426 (37.3 %)	11,857
1990–2004	9937 (92.6 %)	799 (7.4 %)	10,736
Total	17,368	5225	

## Footnotes

1 See, for instance, Andreescu (2013), Broad (1981), Elliott (2013), Kumar (2008), and von Elm et al. (2004) for a compilation of types of misconduct.

2 The author is aware of the irony of this statement.

3 Which might be due to the conditions our institutes subscription.

4
http://www.crossref.org/.

5 Simple code link <p> but also more complex like <xml…>.

6 Theology is meant here not as the study of God(s) but as the science of things divine; “All theology represents an intellectual *rationalization* of the possession of sacred values” (Weber 1970a).
